# Ingleby Lectures: Lecture V

**Published:** 1891-05-09

**Authors:** 


### SURGERY.

### INGLEBY LECTURES.—Lecture V.

Mr. Lloyd in his fifth lecture referred first to Vesical Calculus. Stone in the bladder is much less common in the district of Birmingham than it was fifteen or twenty years ago, and when occurring in young people it is almost entirely confined to the hospital class. Its leading symptoms are: (1) Frequent micturition; (2) pain referred to the end of the penis; (3) the passage of a few drops of blood following micturition; and *in children,* incontinence of urine, prolapsus ani, and a habit of pulling at the prepuce are often the first signs observed, and when consulted concerning any of these conditions one should always bear in mind the possibility of such conditions being due to vesical calculus. With a sound in the bladder under anæsthesia it is easier to say positively that a stone is present than it is to affirm that there is none. The question of *operative treatment* has been much under discussion lately, owing to the revival of the supra-pubic operation, and to the recent application of litholopaxy to young patients. As regards the *supra-pubic operation,* with its recent modifications, this is by far the easiest, and Mr. Lloyd thinks the safest, cutting operation for stone. The modern improvements consist simply in filling the bladder with a known quantity of liquid, and the rectum by means of an india-rubber bag, which is introduced collapsed, and then distended; thus the peritoneum is raised to such a distance above the pubes that there is no risk of injuring it. The great point of the operation is that you can see everything you are cutting through. *Practical points in the operation* are: (1) Distend the bladder until it is felt above the pubes, or till in pushing the piston of the syringe you feel a distinct resistance; (2) by a median incision, cut quickly down to a layer of fat beneath the muscles the sub-peritoneal fat, and then lay bare the pale muscular tissue of the bladder with the handle of the scalpel. (3) Hook up the bladder into a well-curved tenaculum, which must not be withdrawn till the stone is extracted. (4) Puncture the bladder with a scalpel, and enlarge as required by digital dilatation. (5) Remove the stone with a finger and a scoop; this is easier than by forceps. (6) Suture the bladder walls only when the urine is perfectly healthy, and do not let any part of the sutures enter the bladder cavity. (7) Lay a drainage tube down to, but not into, the bladder. There is no need to tie a catheter in the bladder. Stitch the soft parts closely round the tube. Concerning the relative merits of lateral and supra-pubic lithotomy, Mr. Lloyd recommends the high operation in adults when litholopaxy cannot be performed; and also he recommends it in children. Litholopaxy has lately been established as a practical operation in young children with suitable instruments, but has not been sufficiently tried to allow it to supersede lithotomy.

Irritable or Rugose Bladder.—A condition is occasionally met with in children where all the symptoms of stone exist and yet there is no stone. It is said to depend on renal calculus, hyperacidity of the urine, and strumous inflammation.

In the adult many of these cases are due to diseases of the seminal vesicles, and in children the seat of disease is often away from the bladder altogether.

Retention of Urine.—In small boys this nearly always depends on impacted urethral calculus. In such a case you will not be able to pass a catheter. If you cannot extract the stone by the meatus it is best to push it back into the membranous portion of the urethra, and cut down upon it from the perinæum, for then fistula is much less likely to follow than if you incised the penile urethra.

Incontinence of Urine.—Mr. Lloyd has seen several cases where this was due to infantile paralysis.

For the treatment of eneuresis there is no better remedy than belladonna in large doses in combination with boracic acid and biborate of soda.

Phimosis.—This is a cause of suffering at all ages. In the baby it gives rise to urinary incontinence, reflex spasms, convulsions, and to hernia. In the small boy it predisposes to masturbation. In the young man it increases the susceptibility to venereal diseases. In the old man it favours the development of cancer. The best treatment is circumcision, taking care to take enough skin away.

Diseases of the Rectum.—These are by no means rare in children.

*Prolapsus Ani.*—This is the commonest affection of the rectum in children. It is usually symptomatic of intestinal catarrh, worms, phimosis, debility, or vesical calculus. The best local application is one composed of dried sulphate of iron, 8 grains; sulphate of morphia, two grains; and one ounce of spermaceti ointment. The most intractable cases occur in marasmic infants. *Rectal Polypus.*—This always should suggest itself to the mind in young children who suffer from hæmorrhage and tenesmus. It is easily removed after ligaturing the pedicle. *Fissure of the Anus.*—This condition is often met with. It causes much suffering to the patient, and much misconception in the mind of the mother. A child in good health who jibs at the approach of the chamber utensil should be examined for anal fissure. If found, a simple astringent ointment will usually cure; but if the case is obstinate, forcible dilatation of the anus should be employed. *Fistula in Ano.*—In the young, fistulæ are not infrequently the sinuses leading down to dead bone. Mr. Lloyd quoted the case of a boy he was operating on in which the fistulous track ran some distance up the bowel, and finally led to a sequestrum, consisting of half the body of the pubes, which he removed.

Tumours.

Among the tumours peculiar to early life is that variety known as *congenital cystic hygroma.* These tumours are mostly met with in the neck, axilla, sometimes in the limbs and scrotum. They sometimes attain an enormous size, but are not malignant. They are made up of scores of cysts bound together with tough fibrous tissue. They have a characteristic lobulated feel. Not being encapsuled when they are situated near important nerves and blood vessels their enucleation should not be attempted; all you can then do is to puncture the individual cysts which are accessible from the surface. *Tumoid Cyst of the Orbit.*—This is a common congenital tumour and is met with at the outer angle of the orbit. When excising them remember some communicate with the cranial cavity. *Malignant Tumours.*—These are usually sarcomatous and most commonly affect the lower end of the femur or the upper end of the humeris; sometimes they occur in the kidney, and many then attain an enormous size.

